# Supplementary material for: New Technique for Enhancing Residual Oil Recovery from Low-Permeability Reservoirs: The Cooperation of Petroleum Hydrocarbon-Degrading Bacteria and SiO2 Nanoparticles
Source: Microorganisms. 2022 Oct 24;10(11):2104. doi: 10.3390/microorganisms10112104 (PMC9695329; doi:10.3390/microorganisms10112104)
Supplement: Supplementary file 1 [file microorganisms-10-02104-s001.zip › microorganisms-1939242-Supplementary material.pdf]

Supplementary Materials

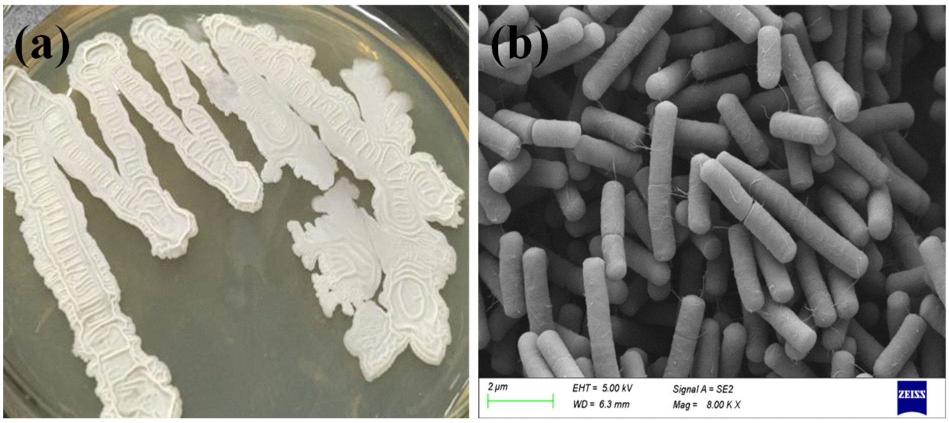

Figure S1. Morphological characteristics (a) and size (b) of PHDB.

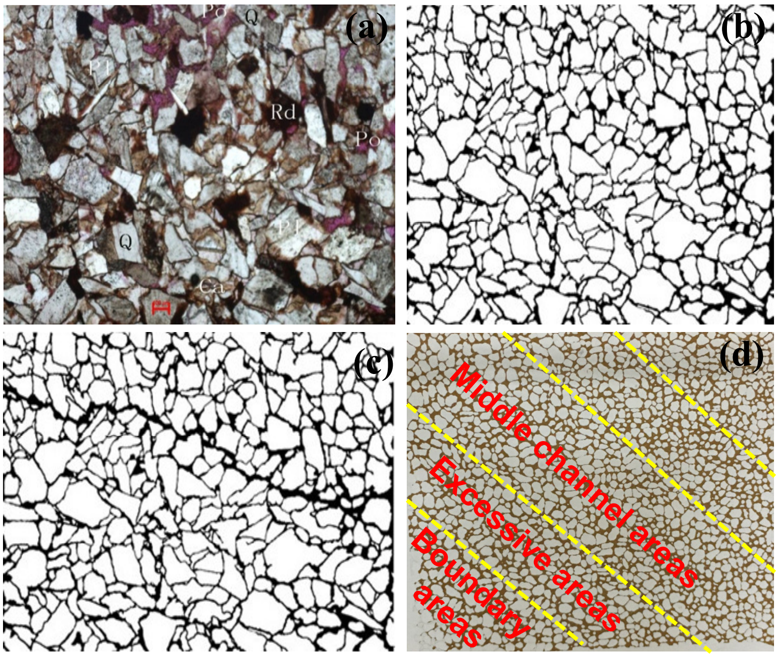

Figure S2. Schematic diagram of the glass etching-micromodel structure. (a) Real core slice, (b) Homogeneous pore structure model, (c) "Fracture-pore" dual medium structure model, and (d) Visual glass micromodel.

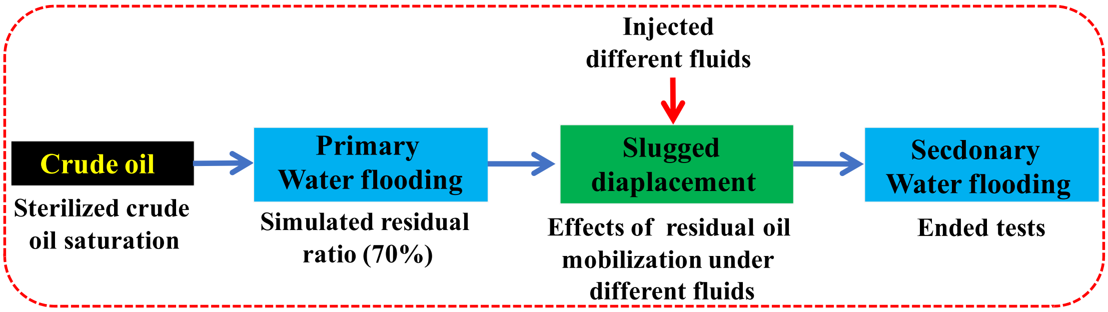

Figure S3. A flow chart of displacement experimental procedures in the micromodels.

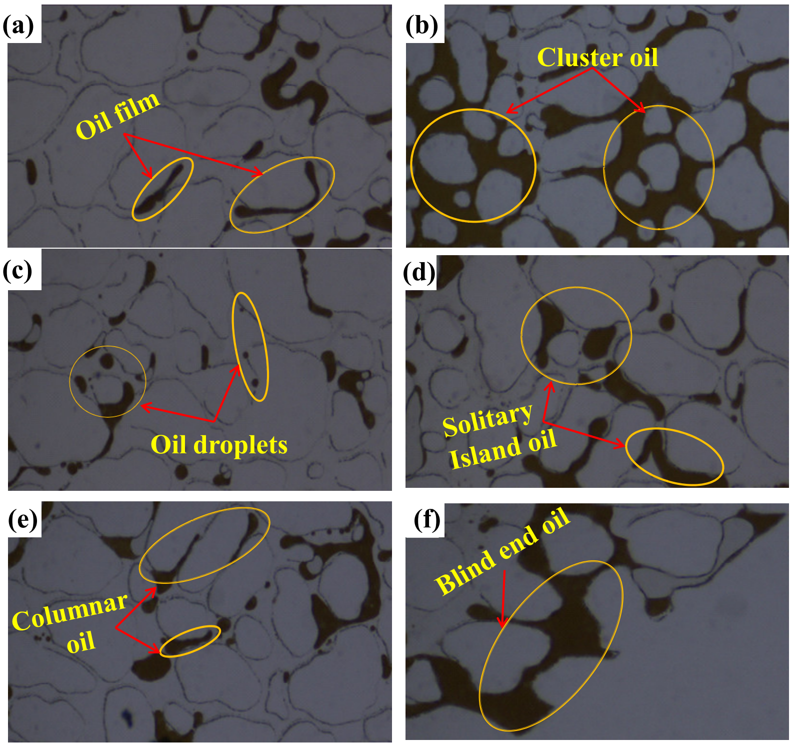

Figure S4. Distribution characteristics of residual oil after primary water flooding. (a) The residual oil in oil film form, (b) The residual oil in cluster form, (c) The residual oil in oil droplets form, (d) The residual oil in solitary island form, (e) The residual oil in columnar form, and (b) The residual oil in blind-end form.

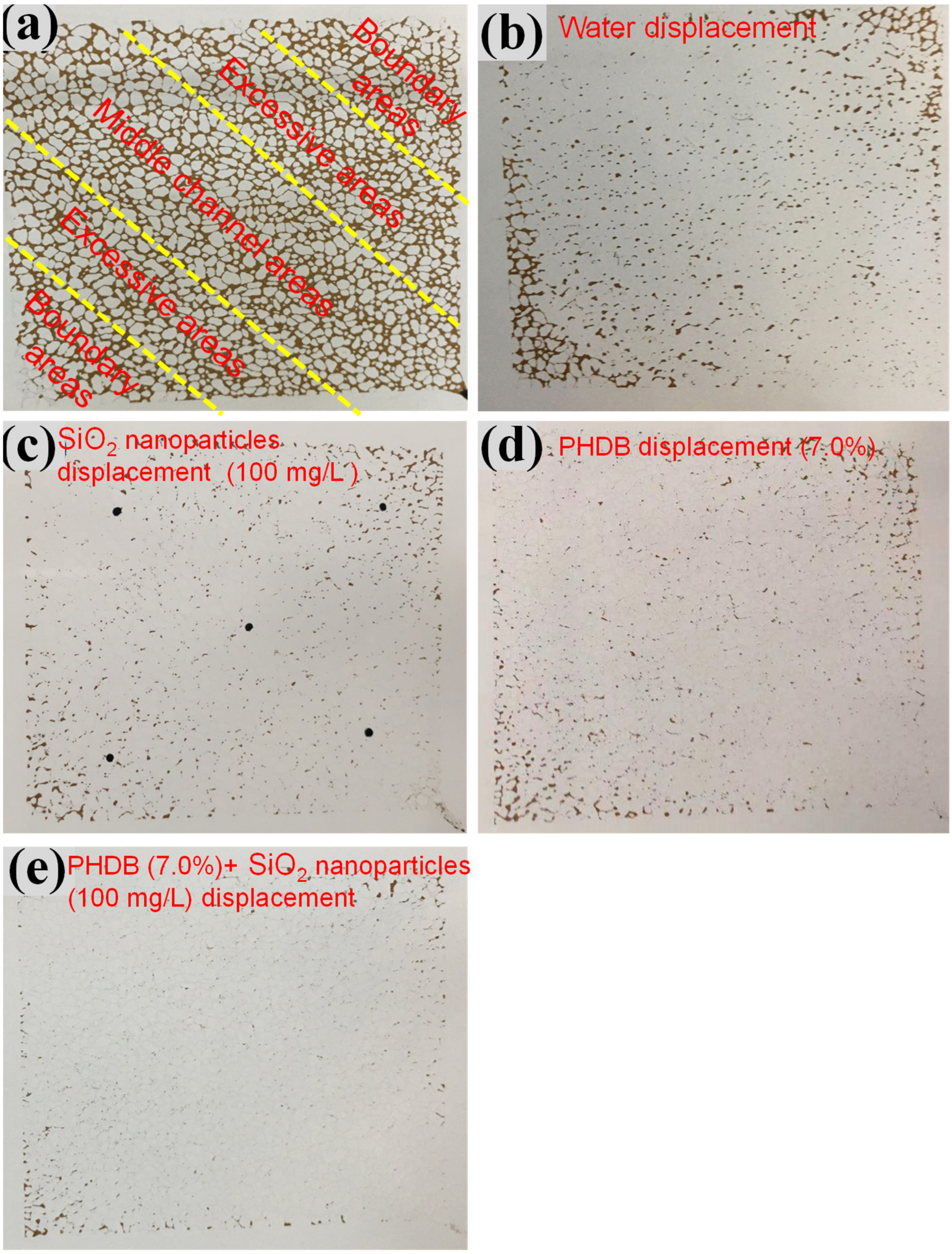

Figure S5. Distribution characteristics of residual oil under different displacement fluids. (a) Saturated oil (blank control group, without water displacement), (b) Secondary water displacement (experimental control group), (c) SiO_2 nanoparticles displacement (100 mg/L), (d) PHDB displacement (7.0%), and (e) PHDB (7.0%)-SiO_2 nanoparticles (100 mg/L) composite displacement.

Table S1. Characteristics of the production water

Property	pH	Salinity (mg/L)	Nitrogen (mg/L)	Phosphorus (mg/L)	Total bacteria (CFU/mL)	PHDB (CFU/mL)
Value	8.13	20000	11.4	24.6	$2.7×10^6$	$7.0×10^2$
